# Microtubule–mitochondrial attachment facilitates cell division symmetry and mitochondrial partitioning in fission yeast

**DOI:** 10.1242/jcs.260705

**Published:** 2023-01-12

**Authors:** Leeba Ann Chacko, Felix Mikus, Nicholas Ariotti, Gautam Dey, Vaishnavi Ananthanarayanan

**Affiliations:** ^1^ Centre for BioSystems Science and Engineering, Indian Institute of Science, Bengaluru 560012, India; ^2^ Cell Biology and Biophysics Unit, European Molecular Biology Laboratory, 69117 Heidelberg, Germany; ^3^ Collaboration for joint PhD degree between EMBL and Heidelberg University, Faculty of Biosciences, 69120 Heidelberg, Germany; ^4^ Centre for Cell Biology of Chronic Disease, Institute for Molecular Bioscience, University of Queensland, Brisbane, QLD 4072, Australia

**Keywords:** Microtubules, Mitochondria, Cell division, Mitochondrial partitioning

## Abstract

Association with microtubules inhibits the fission of mitochondria in *Schizosaccharomyces pombe*. Here, we show that this attachment of mitochondria to microtubules is an important cell-intrinsic factor in determining cell division symmetry. By comparing mutant cells that exhibited enhanced attachment and no attachment of mitochondria to microtubules (Dnm1Δ and Mmb1Δ, respectively), we show that microtubules in these mutants displayed aberrant dynamics compared to wild-type cells, which resulted in errors in nuclear positioning. This translated to cell division asymmetry in a significant proportion of both Dnm1Δ and Mmb1Δ cells. Asymmetric division in Dnm1Δ and Mmb1Δ cells resulted in unequal distribution of mitochondria, with the daughter cell that received more mitochondria growing faster than the other daughter cell. Taken together, we show the existence of homeostatic feedback controls between mitochondria and microtubules in fission yeast, which directly influence mitochondrial partitioning and, thereby, cell growth.

This article has an associated First Person interview with the first author of the paper.

## Teaser

**Summary:** Microtubules and mitochondria cooperate to ensure that a fission yeast cell divides symmetrically and that mitochondria are divided properly between the resulting daughter cells.

## INTRODUCTION

Symmetric cell division is the hallmark of most eukaryotic cells. The fission yeast (*Schizosaccharomyces pombe*) is a rod-shaped, unicellular eukaryote that divides symmetrically during mitosis (Forsburg and Rhind, 2006). A single cell grows by polarised tip extension from about 7 μm to 14 μm in length. Once the cell has grown to 14 μm in length, it ceases to grow and proceeds to divide by assembling an actomyosin contractile ring at the geometric centre of the cell (Piel and Tran, 2009; Lee et al., 2012). Subsequently, the two daughter cells formed post mitosis are of equal length. Owing to its ability to divide medially and produce identically sized daughter cells, fission yeast is a powerful tool in cell cycle research.

One of the key players involved in ensuring symmetric division in fission yeast has been identified to be the microtubule (MT) cytoskeleton (Tran et al., 2001). A typical fission yeast cell contains an average of three to five MT bundles that emanate in the perinuclear region from the centrosome (spindle pole body in yeast) or other interphase MT-organising centres (iMTOCs) (Sawin and Tran, 2006), and are positioned along the long axis of the cell (Höög et al., 2007). MTs in *S. pombe* can crossbridge with the nuclear envelope (Höög et al., 2007) and iMTOCs themselves are thought to interact with the nuclear envelope (Tran et al., 2001). The pushing forces from the individual MT bundles growing against the cell periphery in an interphase cell ensure the medial placement of the nucleus (Tran et al., 2001). This medial placement enables positioning of the division plane at the centre of the cell (Daga and Chang, 2005). As a result, attenuating the dynamics of MTs causes severe cell division defects.

Contrary to their depiction in textbooks, mitochondria are not discrete, static entities, but rather a network of tubules that are in an equilibrium between fission and fusion. This balance between fission and fusion is essential for proper mitochondrial function, with dysfunction being associated with several cellular metabolic defects (Westermann, 2008). The dynamin-related GTPase Drp1 (Dnm1 in yeast) is the major mitochondrial fission protein, whereas the GTPases Mfn1/Mfn2 and Opa1 bring about fusion of the outer membrane and inner membrane of the mitochondria, respectively (Chan, 2006; Mishra and Chan, 2014; Westermann, 2012). Dnm1 is cytosolic but assembles as rings around the mitochondrial outer membrane and undergoes GTP hydrolysis to effect constriction and eventual scission of mitochondria (Ingerman et al., 2005; Mears et al., 2011). In the absence of Dnm1, mitochondria exist as a single, long network that spans the entire cell, but remains attached to MTs (Jourdain et al., 2009).

In fission yeast, mitochondria are bound to MTs via the linker protein Mmb1 (Fu et al., 2011). Recently, we showed that the absence of Mmb1 results in mitochondrial fragmentation owing to the inability of Dnm1 to assemble around mitochondria bound to MTs (Mehta et al., 2019). In cells with MTs shorter than normal, we observed several shorter mitochondria, whereas in cells with MTs longer than wild-type (WT) MTs, we observed fewer, longer mitochondria. Importantly, the total mitochondrial volume between the WT cells and mutant strains with shorter or longer MTs was conserved, indicating that the predominant result of altered MT dynamics was a change in mitochondrial morphology. We therefore established a causal link between MT dynamics and mitochondrial morphology (Mehta et al., 2019).

In this work, we explore the outcome of altered mitochondrial form and the resultant attachment of mitochondria to MTs in the context of cell division. We observed that both Dnm1Δ and Mmb1Δ cells displayed increased asymmetric cell division. We thus investigated the mechanism by which alteration of the mitochondrial form resulted in these cellular homeostasis defects.

## RESULTS

### Dnm1Δ and Mmb1Δ exhibit asymmetry during cell division

**Fig. 1.**
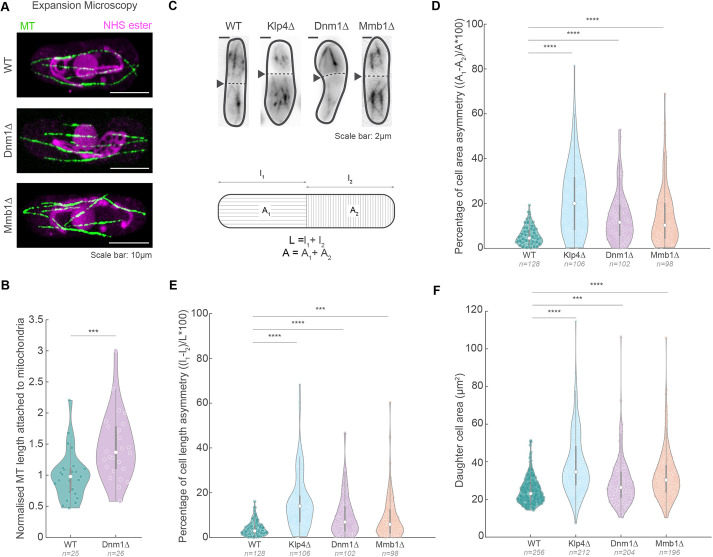
**Dnm1Δ and Mmb1Δ cells exhibit increased asymmetric cell division.** (A) Spinning-disk confocal microscopy images of MTs (green) and NHS ester (magenta) in ultrastructure-expanded WT, Dnm1Δ and Mmb1Δ cells (strains L972, Dnm1Δ and VA078; see Table S1). The NHS ester non-specifically labels protein density, particularly the mitochondria and nucleus, as seen in these cells. (B) Quantification of MT length attached to mitochondria in WT and Dnm1Δ cells normalised to the mean of WT cells (mean±s.d.: 1.0±0.4 and 1.5±0.5 for WT and Dnm1Δ cells, respectively). Note that Mmb1Δ cells do not show attachment between MTs and mitochondria. ***P<10^−3^, two-tailed unpaired Student's *t*-test. (C) Maximum-intensity projected images (top) of MTs in WT, Klp4Δ, Dnm1Δ and Mmb1Δ (strains L972, FY7143, KI001, G5B, Dnm1Δ, and VA069), with the cell division plane (dashed line) indicated by the black arrowheads, and schematic (bottom) of the method employed to measure cell length and area asymmetries. (D) Plot of asymmetry in cell areas between the daughter cells in WT, Klp4Δ, Dnm1Δ and Mmb1Δ cells (mean±s.d.: 5.5±4.0, 21.7±15.5, 13.9±11.3 and 14±13%, respectively). (E) Plot of asymmetry in cell lengths between the daughter cells in WT, Klp4Δ, Dnm1Δ and Mmb1Δ cells (mean±s.d.: 4.0±3.2, 16.1±12.8, 9.9±9.3, and 8.7±9.6%, respectively). (F) Plot of daughter cell area in WT, Klp4Δ, Dnm1Δ and Mmb1Δ cells (mean±s.d.: 24.5±6.9, 38.7±16.2, 28.9±12.0 and 32.5±13.0[Supplementary-material sup1]μm^2^, respectively). For D–F, ***P<2×10^−4^; ****P<10^−4^; Kruskal–Wallis test for non-parametric data. The boxes in B–F represent the 25–75th percentiles, whiskers (1.5× interquartile range) show the most extreme data points not considered outliers and the median is indicated; the shapes of the violin plots represent the kernel density estimate of the data.

Cells lacking the mitochondrial fission protein Dnm1 contain a single long mitochondrial network (Fig. S1A) (Jourdain et al., 2009). This long mitochondrion was attached to MTs along the length of the cell, such that when MTs were depolymerised using methyl-2-benzimidazole-carbamate (MBC), we observed retraction of the mitochondrial network (Fig. S1B; Movie 1). This evinced that there was an enhanced attachment of mitochondria to MTs in Dnm1Δ cells. On the contrary, it has been reported that cells lacking the mitochondria–MT linker protein Mmb1 do not associate with MTs (Fu et al., 2011). We confirmed these observations by visualising MTs and mitochondria in images of ultrastructure-expanded cells (Hinterndorfer et al., 2022) of high-pressure-frozen WT, Dnm1Δ and Mmb1Δ cells (Fig. 1A; Fig. S1A; Movie 2) (Laporte et al., 2022), and indeed quantified higher rates of attachment of mitochondria to MTs in Dnm1Δ cells compared to WT cells (Fig. 1B). In our previous work, we showed that this dissociation of mitochondria from MTs results in fragmentation of the mitochondrial network (Fig. S1A) (Mehta et al., 2019). When we followed dividing Dnm1Δ and Mmb1Δ cells, we observed that cells exhibited ∼15% asymmetry in both cell length and cell area during division, compared to a median of ∼5% asymmetry in WT cells (Fig. 1C–E; Fig. S1C). Accordingly, the daughter cells in the Dnm1Δ and Mmb1Δ background were also distributed across a larger range of areas than the WT cells (Fig. 1F). This degree of asymmetry during division is significantly higher than that seen in WT cells but slightly lower compared to the phenotype in Klp4Δ (where Klp4 or Tea2 is a MT-stabilising kinesin-like protein) (Fig. 1) (Browning et al., 2000) and Pom1Δ (where Pom1 is a polarity-determining protein kinase) (Fig. S1D,E) (Bähler and Pringle, 1998) cells, which have been well-established to exhibit asymmetry in division. Cells lacking the heteromeric kinesin-8 proteins Klp5 and Klp6 (Klp5/6) have longer MTs and mitochondria than WT (Mehta et al., 2019), and therefore also have increased attachment of mitochondria to MTs. Klp5/6Δ cells also showed increased asymmetric division compared to WT cells (Fig. S1D,E).

We asked whether the asymmetry could have arisen due to defects in mitochondrial function in the mutant cells. To answer this question, we quantified the proportion of asymmetry in dividing *rho*^0^ cells. *S. pombe* cells are petite negative, that is, they are inviable in the absence of mitochondrial DNA (mtDNA), and *rho*^0^ cells have an additional nuclear mutation to grow in the absence of mtDNA (Haffter and Fox, 1992; Chacko et al., 2019). These *rho*^0^ cells rely primarily on glycolysis for ATP production, and therefore grow slower on fermentable carbon sources (Haffter and Fox, 1992). We did not observe significant differences in cell division asymmetry between WT and *rho*^0^ cells (Fig. S1D,E). Mitochondrial form is also linked to reactive oxygen species (ROS) levels, with fragmented mitochondria producing increased levels of ROS and fused mitochondria producing reduced levels of ROS (Pletjushkina et al., 2006). However, in our previous work, we did not see a difference in mitochondrial ROS in mutants with altered mitochondrial morphology (Mehta et al., 2019). Similar to previous results (Mehta et al., 2019), transformation of Dnm1Δ cells with Dnm1 restored mitochondrial form and also symmetry in daughter cell length during division (Fig. S1C,D).

### Microtubule dynamics are altered in mitochondrial morphology mutants

**Fig. 2.**
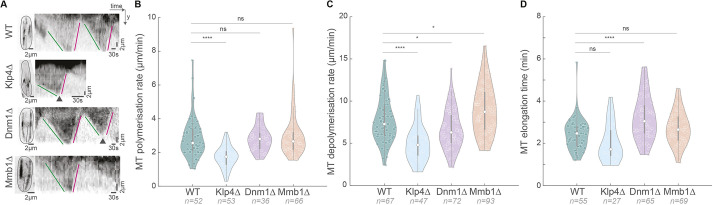
**MT depolymerisation rate is aberrant in Dnm1Δ and Mmb1Δ cells.** (A) Maximum-intensity-projected images (left) of MTs from the first frame of time-lapse videos of representative WT, Klp4Δ, Dnm1Δ and Mmb1Δ cells (strains VA112, G5B, VA110 and VA113; see Table S1), and the corresponding kymographs (right) of the MTs indicated with the square bracket. Green lines indicate MT polymerisation, magenta lines indicate MT depolymerisation and the arrowheads point to catastrophe events. (B) Plot of MT polymerisation rates in WT, Klp4Δ, Dnm1Δ and Mmb1Δ cells (mean±s.d.: 2.9±1.2, 1.7±0.6, 2.8±0.7 and 3.0±1.3 μm/min, respectively). (C) Plot of MT depolymerisation rates in WT, Klp4Δ, Dnm1Δ and Mmb1Δ cells (mean±s.d.: 7.8±2.7, 5.0±2.1, 6.7±2.3 and 9.0±2.9 μm/min, respectively). (D) Plot of MT elongation times in WT, Klp4Δ, Dnm1Δ and Mmb1Δ cells (mean±s.d.: 2.4±0.7, 2.0±0.9, 3.2±1.1 and 2.7±0.8 min, respectively). The reciprocal of the MT elongation time gives the MT catastrophe rate. ns, not significant; *P<11×10^−3^; ****P<10^−4^; Kruskal–Wallis test for non-parametric data and ordinary one-way ANOVA with Dunnett’s post hoc test for parametric data. The boxes in B–D represent the 25–75th percentiles, whiskers (1.5× interquartile range) show the most extreme data points not considered outliers and the median is indicated; the shapes of the violin plots represent the kernel density estimate of the data.

Nuclear positioning in *S. pombe* is effected by pushing forces of growing MTs against the cell poles (Tran et al., 2001). Owing to the paired anti-parallel nature of MT bundles in fission yeast (Sawin and Tran, 2006; Höög et al., 2007), this translates to net equal forces on either side of the cell. Therefore, the nucleus largely remains in the centre of the cell and this central location of the nucleus is essential in dictating the future cell division plane. Fission yeast MT mutants, such as Klp4Δ and Klp5/6Δ, have altered MT dynamics, and therefore contain a nucleus that is not centred, leading to a significant increase in asymmetrically dividing cells (Fig. 1C–E; Fig. S1C–E). We asked whether Dnm1Δ and Mmb1Δ cells displayed asymmetry in cell division due to altered MT dynamics. Mmb1Δ cells have been described to have more dynamic MTs than WT cells, and cells overexpressing Mmb1 exhibit more stable MTs (Fu et al., 2011). Similarly, Dnm1Δ cells required a higher concentration of the MT-depolymerising drug thiabendazole to completely abrogate MTs (Jourdain et al., 2009), indicating higher MT stability. We measured the MT polymerisation rate, depolymerisation rate and MT elongation time in WT, Klp4Δ, Dnm1Δ and Mmb1Δ cells (Fig. 2A), and observed that MTs in Dnm1Δ cells had reduced depolymerisation rates (Fig. 2C) and increased elongation times (reduced catastrophe frequency) compared to WT cells (Fig. 2D). On the contrary, Mmb1Δ cells had MTs with increased depolymerisation rates (Fig. 2C). As expected, Klp4Δ cells exhibited reduced MT depolymerisation rates and polymerisation rates compared to WT cells (Fig. 2A–C). These results indicated that the association of mitochondria with MTs enhanced MT stability, whereas the lack of association reduced MT stability. We confirmed that these results were not an artefact of the levels of tubulin expression in these cells by comparing the total fluorescence intensity of tubulin among the strains employed (Fig. S2A).

### The nucleus is highly dynamic in mitochondrial morphology mutants

**Fig. 3.**
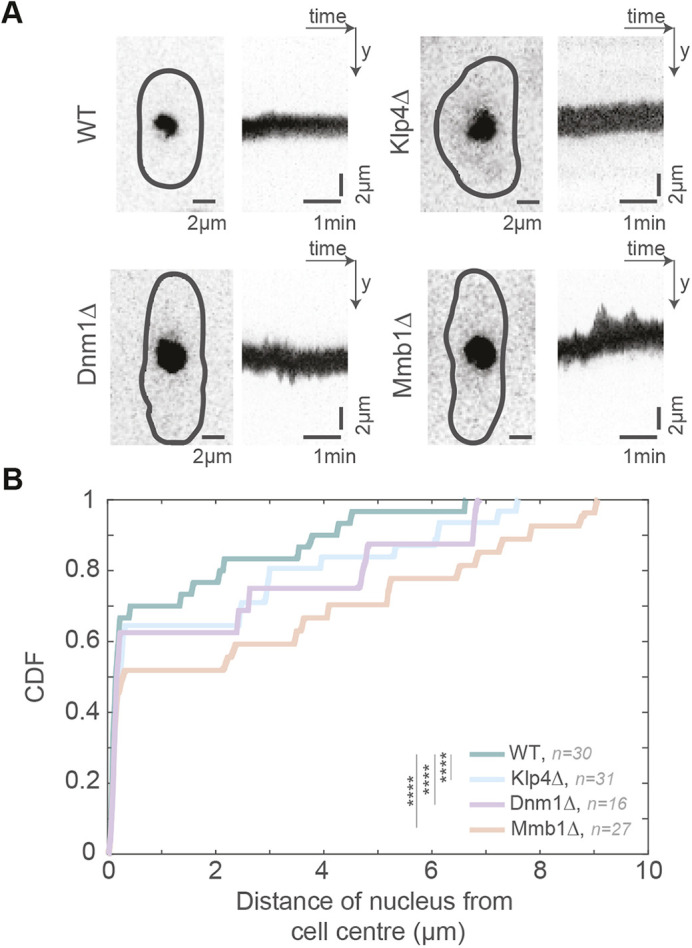
**Dnm1Δ and Mmb1Δ cells exhibit enhanced nuclear movement.** (A) Maximum-intensity-projected images (left) of the nucleus from the first frame of time-lapse videos of representative WT, Klp4Δ, Dnm1Δ and Mmb1Δ cells (strains VA102, VA111, VA103 and VA104; see Table S1), and the corresponding kymographs (right) of the nuclear movement. (B) Cumulative density function (CDF) of the distance of the nucleus from the cell centre for each time point of the time-lapse videos of nuclei in WT, Klp4Δ, Dnm1Δ and Mmb1Δ cells. ****P<10^−4^; Kruskal–Wallis test for non-parametric data.

As the nuclear position prior to the onset of mitosis determines the future site of division (Tran et al., 2001), we asked whether the altered MT dynamics in the mitochondrial morphology mutants changed nuclear dynamics in these cells. We observed that unlike WT cells, the nucleus was highly dynamic in both Dnm1Δ and Mmb1Δ cells (Fig. 3A; Movie 3). As a result, the excursions of the nucleus from the cell centre were significantly higher in Dnm1Δ and Mmb1Δ cells than in WT cells (Fig. 3B). For instance, from the cumulative density function (CDF) plot in Fig. 3B, it can be seen that in 90% of WT cells (0.9 on the *y*-axis), the distance between the nucleus and cell centre was ≤4 μm. However, only about 65% of Mmb1Δ cells (∼0.65 on the *y*-axis) and 75% of Dnm1Δ cells (∼0.75 on the *y*-axis) exhibited the same distance between the nucleus and the cell centre. We confirmed that the nucleus moved more as a result of the altered MT dynamics by visualising the nuclear dynamics in cells devoid of MTs (Fig. S2B). As expected, we measured negligible movement of the nucleus in the absence of MTs. Similarly, it has been reported that the short MTs in Klp4Δ cells typically do not contact the cell end (Browning et al., 2000; Mehta et al., 2019) and therefore do not result in a pushing force to move the nucleus. This was reflected in the reduced movement of the nucleus (Fig. 3A; Movie 3), and increased distance of the Klp4Δ nuclei from the cell centre (Fig. 3B). Occasionally, we observed Dnm1Δ and Mmb1Δ cells that had inherited few or no mitochondria from the mother cell. Remarkably, the nuclei in these cells exhibited dramatic movements (Fig. S2C,D; Movie 4), reiterating the finding that that MT instability could be effected by lack of mitochondrial attachment.

### Mitochondrial partitioning is asymmetric in mitochondrial morphology mutants

**Fig. 4.**
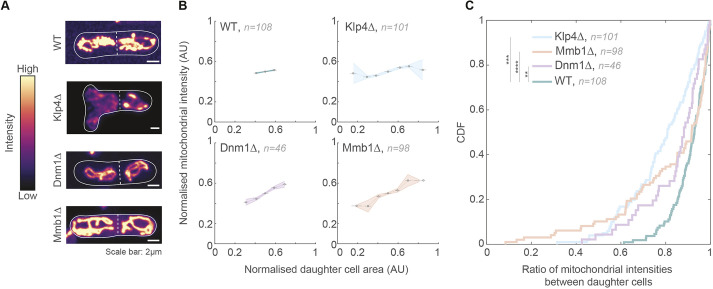
**Mitochondria are asymmetrically partitioned in Dnm1Δ and Mmb1Δ cells.** (A) Maximum-intensity-projected images of mitochondria in WT, Klp4Δ, Dnm1Δ and Mmb1Δ cells (strains KI001, G5B, VA069 and PT2244; see Table S1). Warmer colours indicate higher intensities. The cell outlines are indicated with the solid white line and the septum between the daughter cells is marked with the dashed white line. (B) Plots of normalised mitochondrial intensity (sum intensity) versus normalised cell area in WT, Klp4Δ, Dnm1Δ and Mmb1Δ cells. Data show the mean±s.e.m. AU, arbitrary units. (C) CDF of ratio of mitochondrial intensities between daughter cells in WT, Klp4Δ, Dnm1Δ and Mmb1Δ cells. **P<3×10^−6^; ***P<10^−10^; ****P<10^−21^; Levene's test for equality of variances.

Next, we probed the consequence of asymmetric division of mutant cells on the partitioning of mitochondria. Mitochondria undergo independent segregation in fission yeast, with cell division symmetry aiding the equitable partitioning of mitochondria between daughter cells (Mehta et al., 2019). We measured the mitochondrial intensities in dividing WT and mutant cells (Fig. 4A), and observed that mitochondria were partitioned in proportion to the cell area, indicating that independent segregation was still likely active in the mutants (Fig. 4B). However, as a significant proportion of cells underwent asymmetric division in the mutants, mitochondria were also partitioned unequally between daughter cells (Fig. 4C). For instance, from the CDF plot in Fig. 4C, only about 10% of WT cells (0.1 on the *y*-axis) had a ratio of ≤0.8 between the mitochondrial intensities of the daughter cells (a ratio of 1 implies equal partitioning, and the smaller the ratio, the more unequal the partitioning). On the contrary, ∼48%, 35% and 28% of Klp4Δ, Mmb1Δ and Dnm1Δ cells, respectively, exhibited daughter cell mitochondrial intensity ratios of 0.8 or lower.

### Growth rates of cells scale with the quantity of mitochondria inherited following cell division

**Fig. 5.**
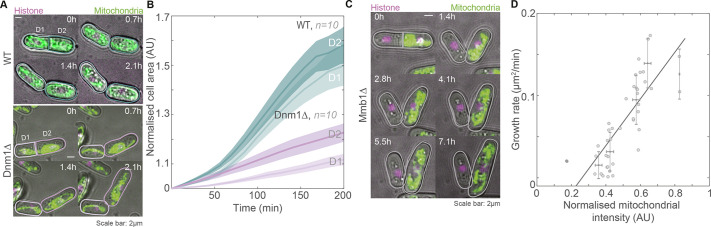
**Mitochondrial content at cell birth determines growth rate.** (A) Montage of maximum-intensity-projected images of mitochondria (green) and histone (magenta) in a representative WT cell (top, strain VA102; see Table S1 for strain details) and Dnm1Δ cell (bottom, strain VA103) undergoing asymmetric cell division and mitochondrial partitioning. D1 is the smaller daughter cell and D2 is the larger daughter cell. (B) Plot of change in cell area of D1 and D2 cells versus time, normalised to the first time frame upon division of the cells. Ten D1-D2 pairs were analysed for WT and Dnm1Δ cells (strains VA102 and VA103). Data show the mean±s.e.m. (C) Montage of maximum-intensity-projected images of mitochondria (green) and histone (magenta) in a representative Mmb1Δ cell (strain VA104) with symmetric cell division but asymmetric mitochondrial partitioning. (D) Plot of growth rate versus mitochondrial intensities in 21 Mmb1Δ daughter-cell pairs that underwent <20% asymmetric cell division. The black line is a weighted linear fit (of the form *y*=*mx*+*c*) and yielded *R*^2^=0.81. AU, arbitrary units. Data show the mean±s.d. AU, arbitrary units.

Finally, we tested the outcome of asymmetric partitioning of mitochondria in Dnm1Δ cells that underwent asymmetric cell division. We observed that the smaller daughter cell, which received a smaller quantity of mitochondria than the larger daughter cell, grew slower than the larger daughter cell (Fig. 5A,B; Fig. S2E,F; Movie 5). In comparison, WT cells, which showed only a small degree of asymmetry in cell area (∼5% on average) and, therefore, in mitochondrial partitioning, still exhibited differences in growth rates between the two daughter cells (Fig. 5B).

We confirmed that the growth rates were proportional to the mitochondria inherited from the mother by quantifying the growth rates in symmetrically dividing cells that partitioned mitochondria asymmetrically. Such events are occasionally seen in Mmb1Δ cells (Fig. 5C,D; Movie 6). We observed a linear relationship between mitochondrial inheritance at the time of birth and the growth rate (Fig. 5D). Furthermore, the growth rate of these cells scaled with mitochondrial concentration and not cell area (Fig. S2G; Jajoo et al. 2016), indicating a central role for mitochondria in determining the dynamics of cell growth.

## DISCUSSION

The interplay between mitochondria and MTs has been implicated in maintaining cellular homeostasis. Here, we first identified that alteration of mitochondrial form and the consequent attachment of mitochondria to MTs resulted in higher rates of incidence of asymmetry in typically symmetrically dividing fission yeast cells. We showed that this asymmetry resulted from changes in MT depolymerisation rate and catastrophe frequency when the association of mitochondria to MTs was either enhanced or absent compared to WT cells. In metazoans, mitochondria rely on microtubules for their transport and positioning (Shah et al., 2021). Furthermore, MTs in metazoans have been demonstrated to effect changes in gene expression owing to their link with the nuclear membrane via the linker of nucleoskeleton and cytoskeleton (LINC) complex (Shokrollahi and Mekhail, 2021). It would be interesting to see whether a change in mitochondrial form or attachment to MTs has a similar effect on MT dynamics and, consequently, on cell fate in metazoans.

The endoplasmic reticulum (ER), another prominent organelle in most cells, has been recently shown to have a mechanical role in controlling MT organisation in mammalian cells (Tikhomirova et al., 2022) and in constraining spindle lengths in *Drosophila* syncytial embryos (Araújo et al., 2022), providing additional evidence for organelle-mediated MT regulation. In *S. pombe*, the ER is not known to directly associate with MTs. However, there might be indirect links between these two components via the ER mitochondria encounter structures, which regulate mitochondrial form and biogenesis (Rasul et al., 2021).

The perturbation of MT dynamics in fission yeast mutants with altered mitochondrial form resulted in increased nuclear movements, which gave rise to nuclear positioning that was offset from the cell centre. As fission yeast relies on nuclear positioning prior to mitosis to dictate the eventual cell division plane, mutants with an altered mitochondrial form exhibited more instances of asymmetric cell division compared to WT cells.

Fission yeast as well as other metazoans have been documented to follow independent segregation to partition mitochondria among daughter cells during mitosis (Mehta et al., 2019; Lawrence and Mandato, 2013). Independent segregation relies on the presence of a large number of mitochondria present in the mother cell to reduce the partitioning error (Huh and Paulsson, 2011). Given large enough numbers of mitochondria, positioning the division plane roughly at the cell centre ensures equitable distribution of mitochondria in daughter cells. In Mmb1Δ and Dnm1Δ cells, owing to the asymmetry observed in a significant proportion of cells, mitochondrial partitioning between the daughters, although equitable, resulted in cells with very few mitochondria compared to the rest of the population. The cells that contained fewer mitochondria were observed to grow slower, and such cells are therefore likely to be outcompeted by other cells. However, because the reduction in mitochondria resulted from altered MT dynamics, asymmetric cell division and consequently daughter cells with fewer mitochondria would persist in future division cycles. Dnm1Δ cells have previously been shown to have retarded growth rates (Dong et al., 2022), which could be partially attributed to the unequal partitioning of mitochondria following asymmetric cell division in a significant proportion of these cells.

Finally, by demonstrating that smaller daughter cells resulting from an asymmetric cell division received less mitochondria and grew slower thereafter, we showed that growth rate scaled with cell size and/or mitochondrial content in the daughter cell. This relationship between mitochondrial content and cell size has been established in mammalian cells and budding yeast (Posakony et al., 1977; Rafelski et al., 2012). However, in fission yeast cells with independent (stochastic) segregation of mitochondria (Mehta et al., 2019), which is the majority of all the cells investigated in this study as quantified in Fig. 4B, the nature of the partitioning mechanism necessitates that mitochondrial content is associated with cell size. Therefore, to delineate whether it was the cell size or the mitochondrial content that defines the growth rate, we quantified the growth rate of daughter cells that had equal cell areas (symmetric cell division) but asymmetric mitochondrial partitioning. These events occur occasionally in Mmb1Δ cells. In this case, we were able to show that the mitochondrial content in the daughter cell at birth was the primary driver of the growth rate. This also implies that the growth rate scaled linearly with the mitochondrial concentration in these cells rather than with cell area. In the future, it would be essential to visualise mtDNA nucleoids to understand whether the growth rate of cells is also influenced by the partitioning of mtDNA in these cells.

In conclusion, MT dynamics and mitochondrial form and attachment were found to be fine-tuned to be in a ‘Goldilocks zone’ in fission yeast, through which symmetric cell division could be achieved. Any deviation from this narrow range resulted in asymmetric cell division. Additionally, cellular homeostasis relied on the feedback between MTs and mitochondria, with the mitochondria dictating their own partitioning via changes in their form. In the future, it will be interesting to understand the fate of cells that inherited fewer mitochondria and whether similar feedback mechanisms exist between the cytoskeleton and other intracellular compartments.

## MATERIALS AND METHODS

### Strains and media

The fission yeast strains used in this study are listed in Table S1. All the strains were grown in yeast extract medium (YES) or Edinburgh minimal medium (EMM) with appropriate supplements at a temperature of 30°C (Forsburg and Rhind, 2006).

### Construction of strains

Strain VA064 was constructed by transforming Dnm1Δ with pREP41-Dnm1 (Dnm1 untagged plasmid; a gift from Isabelle Jourdain, University of Exeter, UK). Similarly, strain VA102 was constructed by crossing PT1650 (h+ cox4-GFP:leu1 ade6-M210 ura4-D18) with JCF4627 (h- ade6-M210 leu1-32 ura4-D18 his3-D1 hht1-mRFP-hygMX6), whereas strain VA103 was constructed by crossing VA077 (h- dnm1::kanr leu1-32ade-(ura+)cox4-GFP:leu1 ade6-M210 leu1-32 ura4-D18) with VA101 (h+ hht1-mRFP-hygMX6 cox4-GFP:leu1 ade6-M210 leu1-32 ura4-D18). Strain VA104 was constructed by crossing VA080 (h- mmb1Δ:Kanr cox4-GFP:leu2 mCherry-atb2:Hygr ade6-m210 leu1-32 ura4-d18) with VA101 (h+ hht1-mRFP-hygMX6 cox4-GFP:leu1 ade6-M210 leu1-32 ura4-D18). Strain VA110 was constructed by crossing VA109 (h+ dnm1Δ::kanr leu1-32ade-(ura+) ura4-Δ18 leu1::GFP-atb2+:ura4+) with JCF4627 (h- ade6-M210 leu1-32 ura4-D18 his3-D1 hht1-mRFP-hygMX6). Strain VA111 was constructed by crossing VA102 (h- hht1-mRFP-hygMX6 cox4-GFP:leu1 ade6-M210 leu1-32 ura4-D18) with MCI438 (h+ tea2d:his3 ade6 leu1-32 ura4-D18 his3-D1). Strain VA112 was constructed by crossing JCF4627 (h- ade6-M210 leu1-32 ura4-D18 his3-D1 hht1-mRFP-hygMX6) with VA106 (h+ ura4-Δ18 leu1::GFP-atb2+:ura4+). Strain VA113 was constructed by crossing VA112 (h+ hht1-mRFP-hygMX6 ura4-Δ18 leu1::GFP-atb2+:ura4+ ade6-M210 leu1-32 his3-D1) with VA078 (h+ mmb1Δ:Kanr). See Table S1 for more details.

### Plasmid transformation

Transformation of strains was carried out using the improved protocol for rapid transformation of fission yeast as described previously (Mehta et al., 2019).

### Preparation of yeast for imaging

For imaging, fission yeast cells were grown overnight in a shaking incubator at 30°C. The following day, the cells were sub-cultured into fresh medium for 2 h at 30°C to achieve an optical density of 0.3–0.4 (mid-log phase). Following this, cells were washed once with distilled water and thrice with EMM. The cells were then allowed to adhere on lectin-coated (Sigma-Aldrich, L2380) 35-mm confocal dishes (SPL Life Sciences, 100350) for 20 min. Unattached cells were removed by washing with EMM.

### Live-cell imaging

Confocal microscopy was carried out in Fig. 1A, Fig. 4A, Fig. S1B and Fig. S2C using the InCell Analyzer-6000 (GE Healthcare) with a 60× air objective, 0.95 numerical aperture (NA) objective fitted with an sCMOS camera. For GFP and RFP imaging, 488 and 561 nm laser lines and 525/20 and 605/52 nm bandpass emission filters, respectively, were used. Spinning-disk confocal microscopy was carried out in Fig. 2A, Fig. 3A and Fig. S1A using the Eclipse Ti2-E (Nikon) with a 100× oil-immersion, 1.49 NA objective fitted with an EMCCD camera (iXon Ultra-897, Andor). For GFP and RFP imaging, 488 and 561 nm laser lines (Toptica) and 525/20 and 605/52 nm bandpass emission filters, respectively, were used.

Laser resonant scanning confocal microscopy was carried out in Fig. 5A,C using the Nikon A1 with a 60×, water immersion, 1.2 NA objective fitted with GaAsP detectors. For GFP and RFP imaging, 488 and 561 nm laser lines and 525/50 and 595/50 nm bandpass emission filters, respectively, with a 405/488/561 nm multi dichroic filter, were used.

MT polymerisation, depolymerisation rates and MT pivoting in Fig. 2B were obtained by imaging *z*-stacks (seven slices with step size 1 μm) acquired every 3 s for 5 min. MT elongation times in Fig. 2D were imaged using *z*-stacks (seven slices with step size 1 μm) acquired every 7 s for 10 min. Short term nuclear dynamics in Fig. 3A were imaged using *z*-stacks (seven slices with step size 1 μm) acquired every 20 s for 20 min, whereas long-term nuclear dynamics in Fig. S2C were imaged using *z*-stacks (five slices with step size 0.5 μm) every 15 min for 12 h. MT depolymerisation in Fig. S1A was observed in time-lapse movies containing *z*-stacks (five slices with step size 0.5 μm) acquired every 12.5 s for 20 min. The growth rates of divided daughter cells in Fig. 5A and Fig. 5C were imaged with *z*-stacks (13 slices with step size 0.5 μm) every 7 min for 10 h and every 14 min for 12 h, respectively.

### Ultrastructure expansion microscopy

Ultrastructure expansion microscopy was performed as described in Hinterndorfer et al. (2022) with some modification to the cell fixation. Briefly, cells were grown in YES at 32°C for 36 h, followed by high-pressure freezing. Cultures were concentrated onto nitrocellulose membranes by vacuum filtration and frozen in 200 μm aluminium carriers in an HPM010 (Abra Fluids, Switzerland). Freeze substitution was performed at −90°C in acetone (Sigma-Aldrich, 24201-M) and gradually warmed to room temperature at 5°C/h. Cells were subsequently rehydrated by successive washes with ethanol containing increasing amounts of H_2_O (0%, 0%, 5%, 5%, 25%, 50% and 100%, 5 min each) and stored until further use in PBS at 4°C (Laporte et al., 2022). For cell wall digestions, fixed cells were rinsed once in PEM buffer (100 mM PIPES, 1 mM EGTA and 1 mM MgSO_4_, pH 6.9) and twice in PEM containing 1.2 M sorbitol (PEMS) before incubating them in 2.5 mg/mL zymolyase 20T (Carl Roth, 9324.3) in PEMS at 37°C with agitation for 45 min. Cell wall digestion was confirmed with Calcofluor White staining (Sigma-Aldrich, 18909), and cells were then washed three times in PEMS buffer. The resulting cell suspension was loaded onto a 12 mm lysine-coated coverslip and processed for expansion.

The coverslips now containing fixed spheroplasts were incubated in protein-crosslinking-prevention solution [2% acrylamide (Sigma-Aldrich, A4058) and 1.4% formaldehyde (Sigma-Aldrich, F8775) in PBS] for 3–5 h at 37°C. To the monomer solution [19% (w/w) sodium acrylate (Sigma-Aldrich, 408220), 10% (w/w) acrylamide, 0.1% (w/w) N,N′-methylenebisacrylamide (Sigma-Aldrich, M1533) in 1× PBS], ammonium persulphate (Thermo Fisher Scientific, 17874) and tetramethylethylenediamine (Thermo Fisher Scientific, 17919) were added at a final concentration of 0.5% each and gelation was performed in a pre-cooled humid chamber on ice for 5 min and at 37°C for 1 h. The coverslips were then incubated in denaturation buffer (50 mM Tris pH 9, 200 mM NaCl and 200 mM SDS in water, pH 9) with agitation for 15 min at room temperature. The formed gels were then transferred to Eppendorf tubes containing denaturation buffer and incubated for 90 min at 95°C without agitation. Gels were expanded by soaking them three times in ddH_2_O for 30 min at room temperature. After full expansion of the gel, the diameter of the gel was measured and processed for immunostaining with N-hydroxysuccinimide (NHS) ester (Thermo Fisher Scientific, 46400; 2 μg/ml in PBS) overnight at 4°C for visualisation of the general organisation of the cell (including mitochondria and the nucleus), and YL1/2 rat anti-α-tubulin antibody (1:25; a gift from Gislene Pereira, Centre for Organismal Studies, Heidelberg, Germany) for the visualisation of MTs. The expanded cells were then imaged using a spinning-disk confocal microscope (Olympus IXplore SpinSR, with 0.95 NA 40× air objective); *z*-stacks spanning the entire cells were taken with a 0.3 μm step size.

### Image and data analysis

Images were analysed using Fiji/ImageJ (Schindelin et al., 2012). Interphase cells that were used in our analyses had a mean length of 10 μm. This mean length corresponds to cells in early-mid G2 phase in *S. pombe* (Nurse, 1975).

For the analysis of the lengths of MTs that were attached to mitochondria in Fig. 1A, the colocalisation (i.e. co-occurrence of fluorescence intensities) of MTs with mitochondria in WT and Dnm1Δ ultrastructure-expanded cells was measured for each step of the *z*-stack containing the entire cell and summed for each cell. The summed values were then normalised to the mean of the WT values.

The MT polymerisation and depolymerisation rates were obtained by measuring the angle of the slopes (*θ*) from kymographs generated by drawing a line along a growing or shrinking MT using the following formula:
(1)

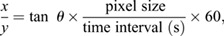
where x is the MT length in micrometres and y is the time in minutes.

The MT elongation time was calculated from the kymograph by measuring the time from the onset of polymerisation to a catastrophe event. The rate of catastrophe was obtained from the reciprocal of the mean elongation time. The nuclear dynamics were obtained by thresholding the nucleus from time-lapse videos in ImageJ to obtain the nuclear centroid, and drawing a region of interest around the cell perimeter to get the cell centroid. Then the Euclidian distance between the two centroids was calculated.

The nuclear velocity in Fig. S2D was determined by measuring the Euclidean distance between the nuclear positions in successive frames. MT pivoting was measured as the difference in the angle of the MT from one frame to another.

For Fig. 4B, the mitochondrial intensities in daughter cells were normalised to the total mitochondrial intensity of the mother and, similarly, the areas of the daughter cells were normalised to the total area of the mother cell just prior to division, such that the sum of the mitochondrial intensities of the two daughter cells and the sum of their areas equalled 1.

For Fig. 5B and Fig. S2E, the cell areas and cell lengths, respectively, were measured in each frame from the first to the last frame and all the cell areas were normalised to the cell area in the first frame. For Fig. 5D and Fig. S2F, the normalised mitochondrial intensity represents the mitochondrial intensities of the daughter cells at birth divided by the mitochondrial intensity of the mother cell. The growth rate represents the rate of change of cell area between the first and last frames of the time-lapse images. Only cells with <20% asymmetry were used for quantification.

### Statistics and plotting

Data were checked for normality using the chi2gof function in MATLAB. Then, to test the statistical significance of the difference between distributions, we used ordinary one-way ANOVA with Dunnett's post hoc test or two-tailed unpaired Student's *t*-test for parametric data and Kruskal–Wallis test or Mann–Whitney test for non-parametric data. Equality of variance was compared using Levene's test. All plots were generated using MATLAB (Mathworks). The figures were organised and prepared in Adobe Illustrator.

## Supplementary Material

10.1242/joces.260705_sup1 Supplementary information
