# Supplementary material for: Microtubule–mitochondrial attachment facilitates cell division symmetry and mitochondrial partitioning in fission yeast
Source: J Cell Sci. 2023 Jan 12;136(1):jcs260705. doi: 10.1242/jcs.260705 (PMC10112971; doi:10.1242/jcs.260705)
Supplement: Supplementary information [file joces-136-260705-s1.pdf]

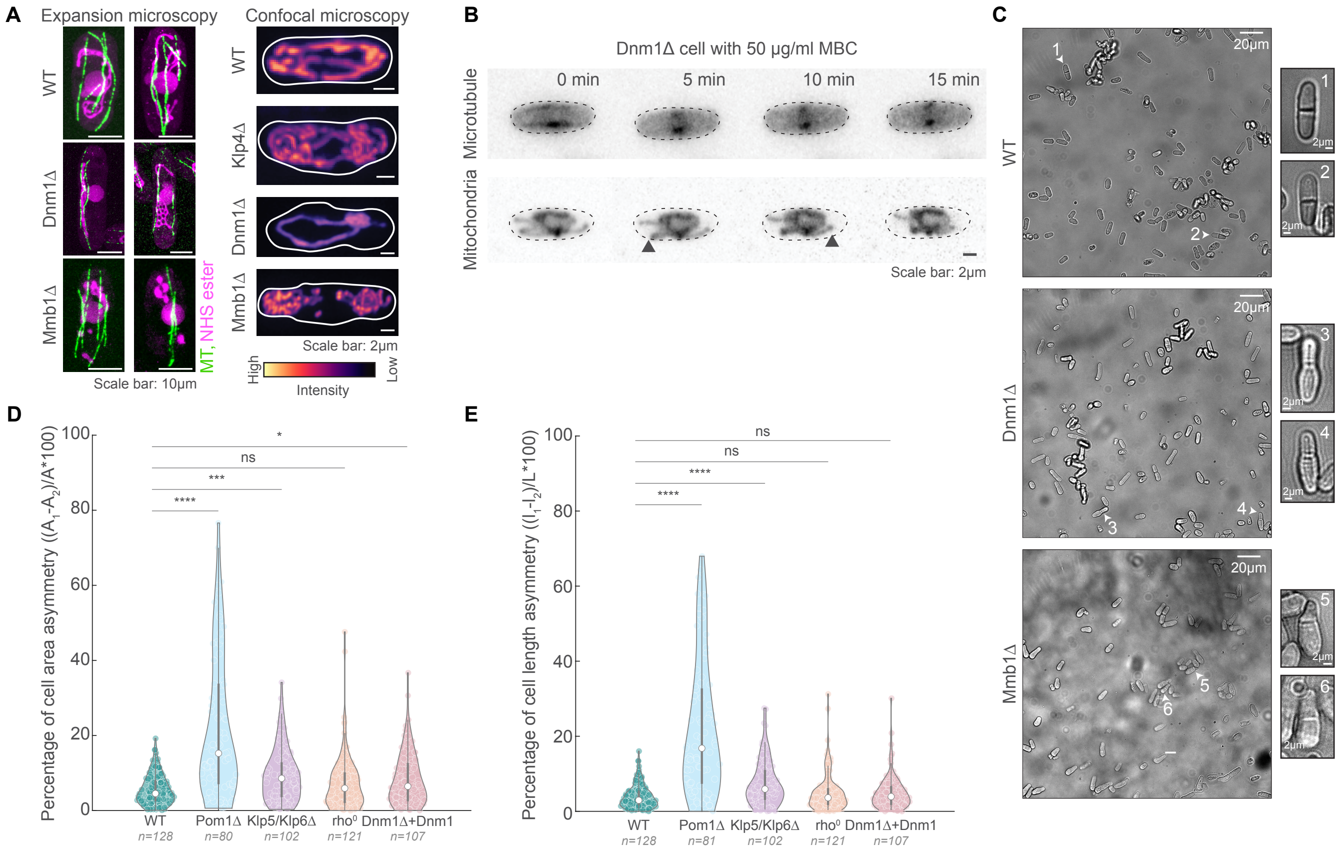

Fig. S1. Pom1Δ and Klp5/6Δ exhibit asymmetric cell division. A, Maximum intensity-projected images of MTs (green) and mitochondria (magenta) in WT, Klp4Δ, Dnm1Δ cells imaged using ultrastructure expansion microscopy (left) and of mitochondria imaged using confocal microscopy (right) in WT, Klp4Δ, Dnm1Δ and Mmb1Δ cells (strains VA102, VA111, VA103, VA104, see Table S1), represented in the intensity map located to the bottom of the images. **B**, Montage of maximum intensity-projected images of MTs and mitochondria in a representative Dnm1Δ cell (strain VA069,see Table S1) treated with 50µg/ml MBC. The change in position of mitochondria is indicated with the grey arrowhead at times 5 and 10 min. We noticed this phenomenon in 100% of the cells observed (n=50). **C**, Representative bright-field images of the whole field of view of WT (top), Dnm1Δ (middle) and Mmb1Δ (bottom) cells. The white arrowheads point to examples of dividing cells which are represented as insets on the right. **D**, Plot of asymmetry in cell areas between the daughter cells in WT, Pom1Δ, Klp5/6Δ, *rho*^{0} and Dnm1Δ+Dnm1 plasmid cells (mean±S.D.: 5.5±4.0, 22.0±18.7, 9.4±7.1, 7.3±7.3, and 14.1±13.0% respectively; strains L972, FY7143, KI001, FY31851, G3B, PHP14 and VA064, see Table S1). **E**, Plot of asymmetry in cell lengths between the daughter cells in WT, Pom1Δ, Klp5/6Δ, *rho*^{0} and Dnm1Δ+Dnm1 plasmid (mean±S.D.: 4.0±3.2, 21.8±18.2, 7.4±6.1, 5.0±5.1, and 5.0±4.6% respectively; strains L972, FY7143, KI001, FY31851, G3B, PHP14 and VA064, see Table S1).

Journal of Cell Science • Supplementary information

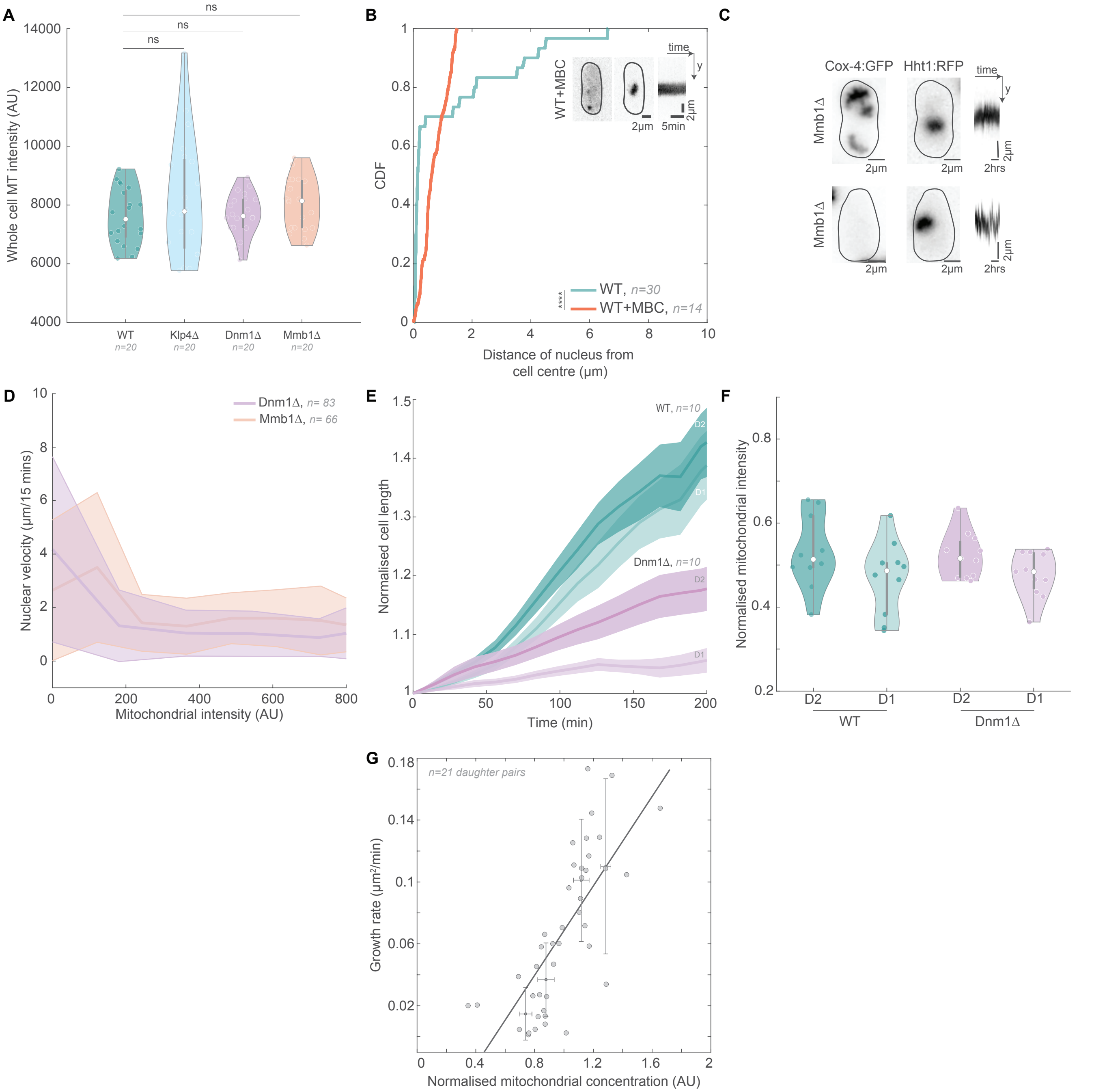

Fig. S2. The nucleus is more dynamic when mitochondria are present in low numbers. A, Plot of summed intensity of the MT in whole WT, Klp4Δ, Dnm1Δ and Mmb1Δ cells (mean±S.D.: $7.6 \times 10^3 \pm 9.4 \times 10^2$, $8.0 \times 10^3 \pm 2.0 \times 10^3$, $7.7 \times 10^3 \pm 7.2 \times 10^2$, and $8.0 \times 10^3 \pm 8.8 \times 10^2$ AU respectively; strains VA112, G5B, VA110, and VA113, see Table S1). 'ns' indicates no significant difference using ordinary one-way ANOVA for parametric data. **B**, CDF of the distance of the nucleus from the cell centre for each time point of the time-lapse videos of nuclei in WT cells and WT cells with 50μg/ml MBC. Note that the WT data has been reproduced from Fig. 4B. Maximum intensity-projected images (top right) of depolymerised MTs and histone in WT+MBC cells

Journal of Cell Science • Supplementary information

(strain VA102, see Table S1). The asterisks (****) represent $p<10^{-4}$, Mann-Whitney Test for non-parametric data. **C**, Maximum intensity-projected images of mitochondria (left) and nucleus (centre) from the first frame of time-lapse videos of representative Mmb1Δ cells (strain VA104, see Table S1). Kymograph (right) depicts nuclear movement from the first to last frame. **D**, Plot of nuclear velocity measured every 15 minutes versus the summed intensity of the mitochondria in Dnm1Δ and Mmb1Δ (strains VA103 and VA104, see Table S1). **E**, Plot of change in cell length vs. time of smaller (D1) and larger (D2) cells normalised to the first time frame upon division of the mother cell. 10 D1-D2 pairs were analysed for WT and Dnm1Δ cells (strains VA102 and VA103, see Table S1). Please note that these data are obtained from the same cells analysed in Fig. 5B. **F**, Plot of normalised mitochondrial intensities of the larger and smaller daughter cells (D2 and D1) in WT and Dnm1Δ cells undergoing asymmetric cell division (mean ±S.D.: 0.53±0.09, 0.47±0.09, 0.52±0.05, and 0.48±0.05 respectively). Please note that these data are obtained from the same cells analysed in Fig. 5B. **G**, Plot of growth rate (change in cell area over time) vs. normalised mitochondrial concentration (ratio of normalised mitochondrial intensity over normalised cell area) in 21 Mmb1Δ daughter cell pairs that underwent symmetric cell division (<20% asymmetry between daughters). The black line is a weighted linear fit (of the form y = mx + c), and yielded R^2 =0.81. Note that these data are obtained from the same cells analysed in Fig. 5D.

Journal of Cell Science • Supplementary information

Table S1.

Name	Genotype	Source
L972	h- WT	Iva Tolic'
FY7143	h- ura4-D18 leu1-32 ade6-M216 his7-366	YGRC, Japan
KI001	h+ sid4-GFP::kan r kan r -nmtP3-GFP-atb2+ nmt1-pCOX4-RFP::leu1+ ura4-D18 ade6-M210	Iana Kalinina
G5B	h- klp4::kanr nmt1-GFP-atb2 leu ura	Rafael Carazo Salas, UK
FY31851	h- leu1-32 CRIB:GFP(ura+) pom1::ura4+ rga4:RFP(kanMX6)	YGRC, Japan
Dnm1Δ	h- dnm1::kanr leu1-32ade-	Yannick Gachet, Toulouse
PT2244	h+ mmb1Δ:Kanr cox4-GFP:leu2 mCherry- atb2:Hygr ade6-m210 leu1-32 ura4-d18	Phong Tran, USA
PT1650	h+ cox4-GFP:leu1 ade6-M210 ura4-D18	Phong Tran, USA
JCF4627	h- ade6-M210 leu1-32 ura4-D18 his3-D1 hht1-mRFP-hygMX6	Julie Cooper Lab
MCI438	h+ tea2d:his3 ade6 leu1-32 ura4-D18 his3-D1	Iva Tolić, Croatia
PHP 14	h- ade6-M216, leul-32, ptp-1, [rho0]	Thomas D. Fox
VA064	h- pREP41-Dnm1(leu+) dnm1::kanr leu1-32 ade-(ura+)	This study
VA069	h- pREP1-atb2:GFP(leu+) dnm1::kanr leu1-32 ade-(ura+))	This study
VA077	h- dnm1::kanr leu1-32ade-(ura+) cox4-GFP:leu1 ade6-M210 leu1-32 ura4-D18	This study
VA078	h+ mmb1Δ:Kanr	This study
VA080	h- mmb1Δ:Kanr cox4-GFP:leu2 mCherry-atb2:Hygr ade6-m210 leu1-32 ura4-d18	This study
VA101	h+ hht1-mRFP-hygMX6 cox4-GFP:leu1 ade6-M210 leu1-32 ura4-D18	This study
VA102	h- hht1-mRFP-hygMX6 cox4-GFP:leu1 ade6-M210 leu1-32 ura4-D18	This study
VA103	h- hht1-mRFP-hygMX6 dnm1::kanr cox4-GFP:leu1 ade6-M210 ura4-D18	This study
VA104	h- hht1-mRFP-hygMX6 mmb1Δ:Kanr cox4-GFP:leu1 ade6-M210 ura4-D18	This study
VA106	h+ ura4-Δ18 leu1::GFP-atb2+:ura4+	This study
VA109	h+ dnm1Δ::kanr leu1-32ade-(ura+) ura4-Δ18 leu1::GFP-atb2+:ura4+	This study
VA110	h- dnm1Δ::kanr leu1-32ade-(ura+) ura4-Δ18 leu1::GFP-atb2+:ura4+ hht1-mRFP-hygMX6	This study
VA111	h- tea2d:his3 hht1-mRFP-hygMX6 cox4-GFP:leu1 ade6-M210 ura4-D18	This study
VA112	h+ hht1-mRFP-hygMX6 ura4-Δ18 leu1::GFP-atb2+:ura4+ ade6-M210 leu1-32 his3-D1	This study
VA113	h+ mmb1Δ:Kanr hht1-mRFP-hygMX6 ura4-Δ18 leu1::GFP-atb2+:ura4+ ade6-M210 leu1-32 his3-D1	This study
pREP41-Dnm1 (plasmid)	Dnm1 (untagged)	Isabelle Jourdain, UK

Journal of Cell Science • Supplementary information

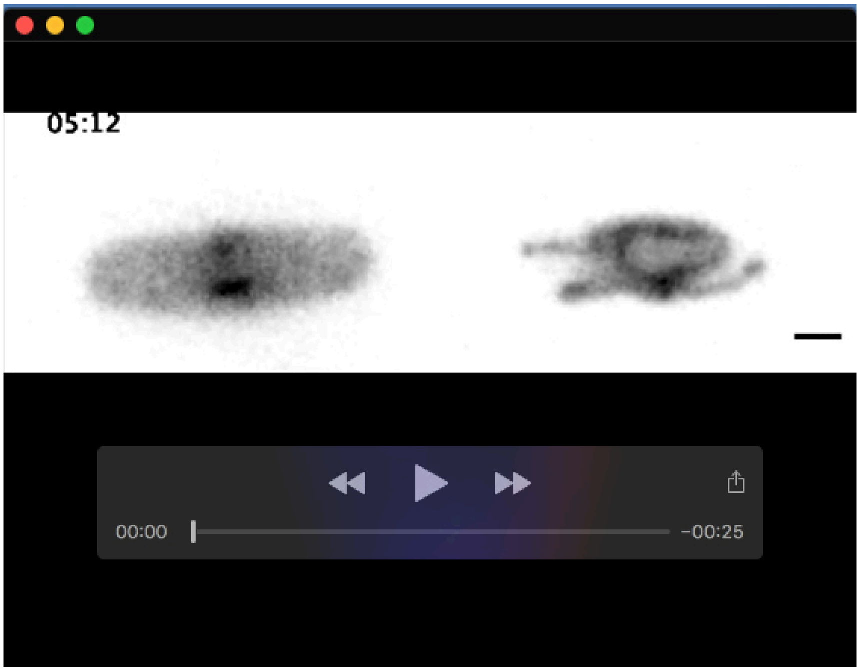

Movie 1. Mitochondria in a Dnm1Δ cell retracts upon addition of MBC.
A representative Dnm1Δ cell stained with 200nM MitoTracker Orange and expressing atb2:GFP (strain VA069, see Table S1) imaged using confocal microscopy every 12.5 s. Scale bar: 2µm, Time is indicated in mm:ss.

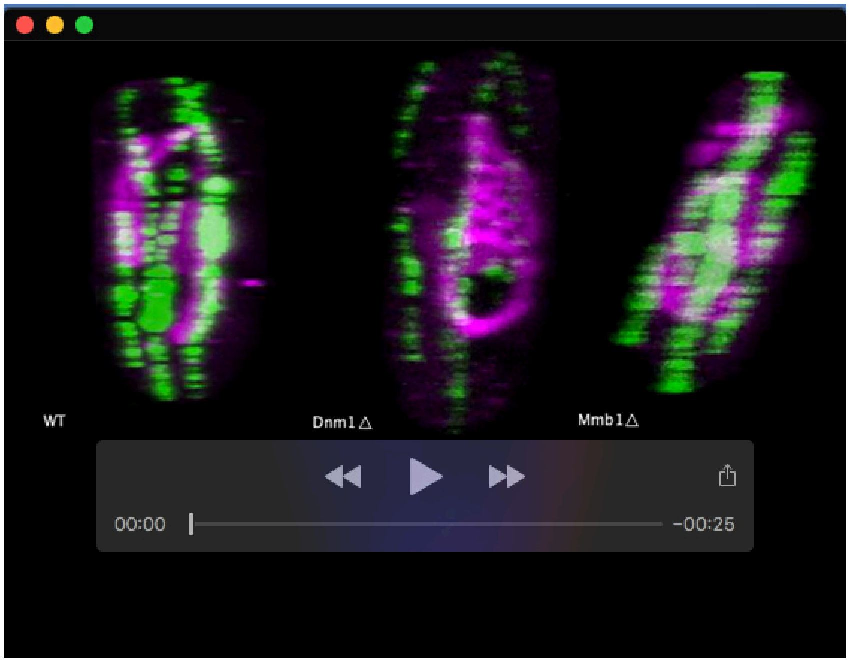

Movie 2. Ultrastructure expansion microscopy reveals increased attachment of mitochondria to MTs in Dnm1Δ cells.
3D projections of representative WT, Dnm1Δ and Mmb1Δ cells stained for the MTs (green) and mitochondria/nucleus (strains L972, Dnm1Δ and VA073, see Table S1) visualised using a spinning disk confocal microscope following ultrastructure expansion.

Journal of Cell Science • Supplementary information

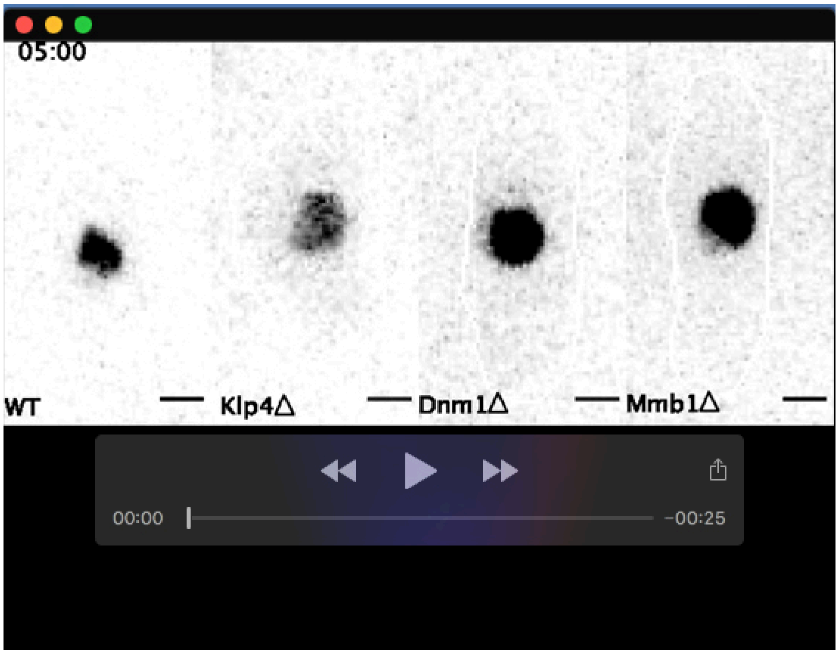

Movie 3. The nucleus is more dynamic in Dnm1Δ and Mmb1Δ cells.
WT, Klp4Δ, Dnm1Δ and Mmb1Δ cells expressing Hht1-mRFP (strain VA102, VA103, VA104 and VA111, see Table S1) imaged using spinning disk microscopy every 20 s. Scale bar: 2μm, Time is indicated in mm:ss.

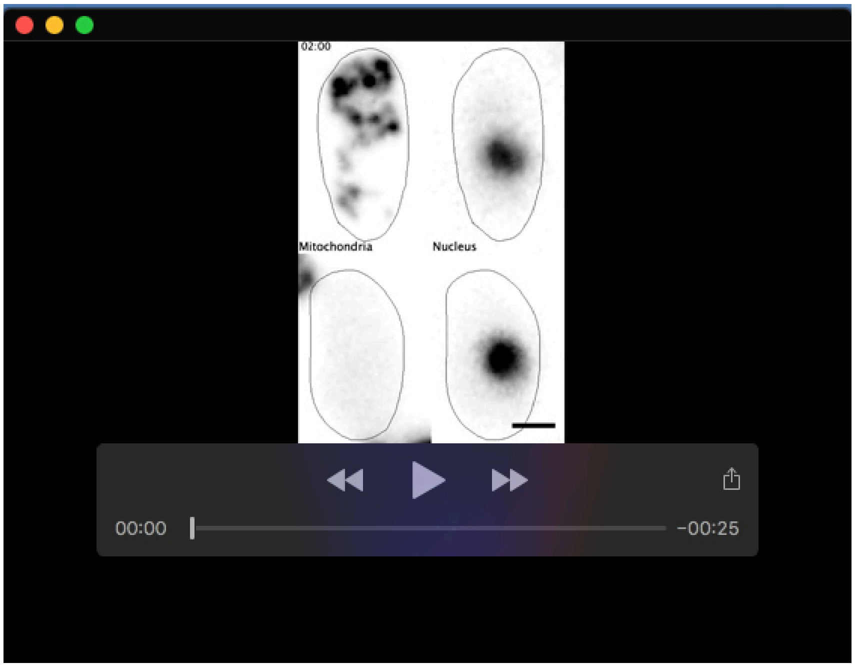

Movie 4. Nuclear dynamics is increased when mitochondria are present in low amounts.
An Amb1Δ Aell Axpressing Aox4:GFP And Aht1:mRFP Astrain AA104, Aee Aable A1) Amaged Asing Aonfocal Aicrosocpy every A5 Ain. Acale Aar: Aμm, Aime As Andicated An Ah:mm.

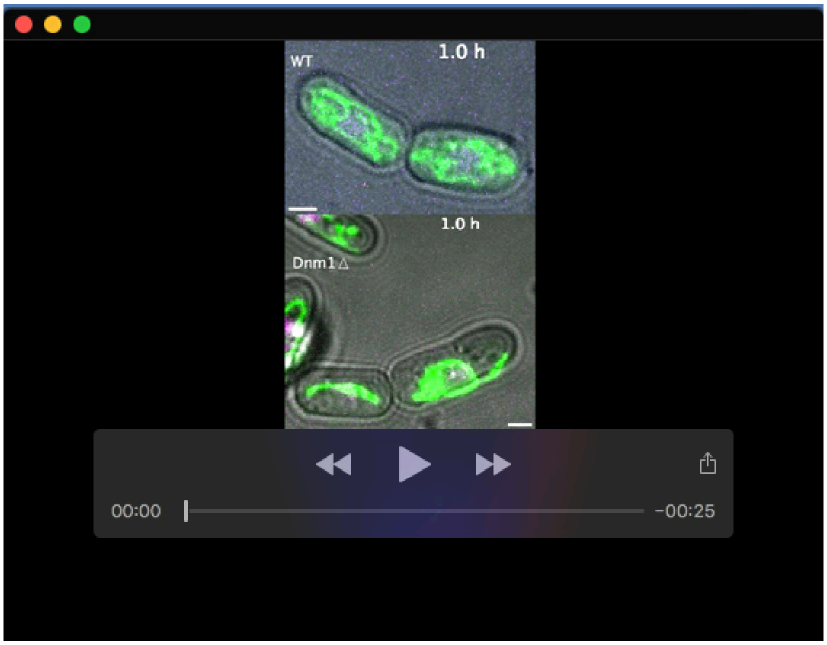

Movie 5. The larger daughter cell grows faster than the smaller one after the mother cell divides asymmetrically.
A WT (top) and Dnm1∆ (bottom) cell expressing expressing Cox4:GFP and Hht:mRFP (strain VA102 and VA103 respectively, see Table S1) imaged using confocal microscopy every 7 min. Scale bar: 2µm.

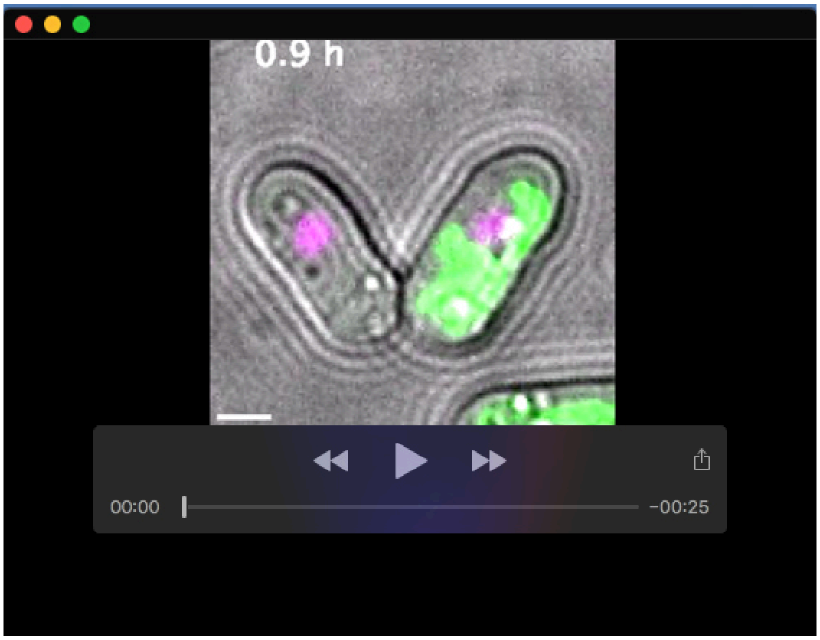

Movie 6. Growth rate of cells scales with the amount of mitochondria inherited at birth.
An Mmb1∆ cell expressing Cox4:GFP and Hht1:mRFP (strain VA104, see Table S1) imaged using confocal microscopy every 14 min. Scale bar: 2µm.

Journal of Cell Science • Supplementary information
